# Quantifying pathogen infection risks from household laundry practices

**DOI:** 10.1111/jam.15273

**Published:** 2021-09-18

**Authors:** Kelly A. Reynolds, Marc P. Verhougstraete, Kristina D. Mena, Syed A. Sattar, Elizabeth A. Scott, Charles P. Gerba

**Affiliations:** ^1^ The Mel and Enid Zuckerman College of Public Health University of Arizona Tucson AZ USA; ^2^ School of Public Health The University of Texas Health Science Center at Houston El Paso TX USA; ^3^ Faculty of Medicine University of Ottawa Ottawa ON Canada; ^4^ Center for Hygiene and Health, Department of Biology Simmons University Boston MA USA; ^5^ Department of Environmental Sciences University of Arizona Tucson AZ USA

**Keywords:** contaminated textiles, hygiene, infections, laundry, pathogens, QMRA, risk reduction

## Abstract

**Aims:**

Contaminated laundry can spread infections. However, current directives for safe laundering are limited to healthcare settings and not reflective of domestic conditions. We aimed to use quantitative microbial risk assessment to evaluate household laundering practices (e.g., detergent selection, washing and drying temperatures, and sanitizer use) relative to log_10_ reductions in pathogens and infection risks during the clothes sorting, washer/dryer loading, folding and storing steps.

**Methods and Results:**

Using published data, we characterized laundry infection risks for respiratory and enteric pathogens relative to a single user contact scenario and a 1.0 × 10^−6^ acceptable risk threshold. For respiratory pathogens, risks following cold water wash temperatures (e.g. median 14.4℃) and standard detergents ranged from 2.2 × 10^−5^ to 2.2 × 10^−7^. Use of advanced, enzymatic detergents reduced risks to 8.6 × 10^−8^ and 2.2 × 10^−11^ respectively. For enteric pathogens, however, hot water, advanced detergents, sanitizing agents and drying are needed to reach risk targets.

**Significance and Impact of the Study:**

Conclusions provide guidance for household laundry practices to achieve targeted risk reductions, given a single user contact scenario. A key finding was that hand hygiene implemented at critical control points in the laundering process was the most significant driver of infection prevention, additionally reducing infection risks by up to 6 log_10_.

## INTRODUCTION

Home laundering is considered a ‘critical control point’ for preventing the spread of infections and maintaining a clean and healthy household (Bloomfield et al., 2011; Bockmühl et al., 2019). Clothing, cleaning tools (e.g. cleaning cloths) and linens readily become contaminated with bodily fluids, dirt and food debris that can contain—and/or become—food sources for pathogenic bacteria, fungi and viruses. Clothing may be contaminated with a wide range of pathogens and serve as vehicles in their transmission. The COVID‐19 pandemic requires assurance that laundering practices are adequate to control the transmission of SARS‐CoV‐2 and other pathogens that may be present in laundry. It has been shown that respiratory, enteric and dermal pathogens can be expected to be present in clothing of ill individuals and those who care for them.

Household laundering has evolved with developments in innovation in the control of the washing machine operations (washwater temperatures, wash cycle times, etc.), design (front vs. top loading), changes in the chemistry of laundry detergents and types of laundry additives, and methods of drying (electric dryers vs. air drying). This has resulted in increased convenience and opportunities to better control the microbial quality of laundry, both in terms of pathogenic and odour‐causing microorganisms (Bockmühl, 2017).

Most of the available literature on pathogen reduction during laundering is focused on healthcare settings where laundry sanitizers are frequently used, or on European wash conditions, where higher water temperatures (e.g. ≥60℃) and longer wash cycles are common. European wash temperatures are generally higher due to the availability of internal heating elements and thermostats in the washing machines that allow users to select higher temperatures. In contrast, wash temperatures in North American machines are generally dependent on incoming water temperatures controlled via the household hot water tank that are often set at relatively lower temperatures due to scalding concerns from tap water feeds.

Hot water washes, however, demand more energy and sanitizers may accelerate damage of many types of fabrics, thus making such practices less desirable from the perspectives of energy conservation and durability of clothing. The objective of this study was to use a quantitative microbial risk assessment (QMRA) approach to compare the impact of variable laundry practices, including lower wash water temperatures (e.g. <40℃), detergents with and without sanitizers, and drying on pathogen survival and infection risks within a single user contact scenario. Additional objectives were to utilize the QMRA approach to provide domestic laundering guidelines for situations of potentially increased risk, such as when household members are ill or laundry is heavily soiled, such as with healthcare personnel uniforms.

### Evidence for transmission of infections via laundry

Epidemiological studies have suggested the role of fabrics in the transmission of infections (Table 1). These studies primarily relate to textiles in healthcare, although childcare settings and nursing homes are also involved. One study suggested the spread of respiratory illness associated with laundromat usage and not using chlorine bleach during laundering (Larson & Duarte, 2001). Outbreaks have been associated both with sharing of items (towels) but also with the survival of pathogens and cross‐infection when processing laundry using inadequate procedures (bed linens) (Bloomfield et al., 2011). Viable SARS‐CoV‐2 viruses have also been isolated from symptomatic patient bedsheets (Ahn et al., 2020). While epidemiological studies show a possible link with exposure, validating transmission risks have been complicated by difficulties in experimental design and sensitivity limits, control of variables, costs and potential confounders of additional pathogen exposure routes.

**TABLE 1:** Outbreaks of pathogens associated with textiles

Microorganism	References
*Salmonella typhimurium*	Steere et al. (1975)
*Salmonella hadar*	Standaert et al. (1994)
*Microsporum canis*	Shah et al. (1988)
*Sacroptes scabiei*	Fijan and Turk (2012)
Acinetobacter	Weernink et al. (1995)
MRSA	Bloomfield et al. (2011)
*Staphylococcus* spp.	Payne (1959)
*Bacillus cereus*	Fijan and Turk (2012)
*Clostridioides difficile*	Owen and Laird (2020)
Hepatitis A virus	Keeffe (2004)
Vaccinia—smallpox	England (1982)
Respiratory infections	Bloomfield et al. (2011)
*Neisseria gonorrhoea*	Goodyear‐Smith (2007)
Hepatitis B virus	Kim and Ahn (1993)

Quantitative microbial risk assessment provides another tool to assess both probabilities of infection and how interventions may reduce the risk of infection by a specific exposure (Haas et al., 2014). This approach has been used to assess the risk of infection from rotaviruses from laundering and has shown that the probability of infection can be as low as 1:10 (Gerba, 2001).

### Occurrence of pathogens in laundry

Most of the microorganisms associated with clothing are from human skin. Other sources include bodily secretions/excretions, food and aerosols. The occurrence of pathogens in laundry has been reviewed in several recent articles (Bloomfield et al., 2011; Bockmühl, 2017; Fijan & Turk, 2012). Viruses, in general, probably present the greatest risk in contaminated textiles because of their greater infectivity (fewer numbers have a greater probability of causing an infection) than bacterial and fungal microorganisms. A wide variety of viruses and other types of pathogens have been detected in textiles representing blood, respiratory, enteric and dermal contamination sources (Table 2).

**TABLE 2:** Viral pathogens detected in laundry

Virus	Reference
Rotavirus	Fijan and Turk (2012)
Hepatitis A	Keeffe (2004)
Papillomavirus	Bergeron et al. (1990)
Hepatitis B	Bloomfield et al. (2011)
Adenovirus	Russell et al. (2006); Da Silva et al. (2014)
Rhinovirus	Gralton et al. (2015)
Influenza	Phan et al. (2019)
Coronavirus	Ahn et al. (2020)
Parainfluenza (assumed)	Phan et al. (2019)
RSV (assumed)	Phan et al. (2019)

### Survival of pathogens in laundry

Survival of pathogens in/on articles of laundry depends on factors such as relative humidity (RH), air temperature and the material type (Yeargin et al., 2016). Most microbial inactivation occurs during drying of the body fluid containing the pathogen (e.g. saliva, mucus) with a subsequent slowing of the rate of inactivation. Even room temperature drying of respiratory viruses results in a usual 10‐ to 100‐fold reduction in the viability titre (Harbourt et al., 2020; Kratzel et al., 2020). SARS‐CoV‐2 survived less than 8 h on clothing at 22 and 37℃, and at least 96 h at 4℃ (Harbourt et al., 2020). In another study, the viability of SARS CoV‐2 was reduced by 99% within 2 h and greater than 99.99% by 48 h on clothing (Chin et al., 2020). Some microorganisms survive better at certain RH than others. The type of material and presence of dyes or colouring agents may also affect persistence as some dyes may be anti‐microbial. The presence of organic matter in heavily soiled textiles may also act to prolong survival or, in the case of some bacteria that can utilize the organic soils as a food source, growth may occur.

Pathogenic bacteria—such as *Salmonella* and MRSA—and moulds, may survive for weeks in clothing (Bloomfield et al., 2011; Kampf, 2020). Most respiratory viruses, including SARS CoV‐2, do not survive more than a day or two in clothing at room temperature (Bean et al., 1982; Ikeda et al., 2015). However, some enteric viruses, such as rotavirus and hepatitis A virus, may survive for several weeks (Boone & Gerba, 2007; Yeargin et al., 2016).

### Steps in laundering

**FIGURE 1:**
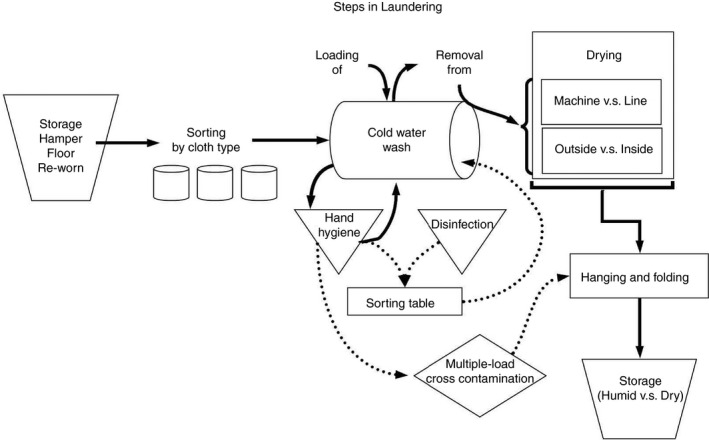
Steps in laundering

Laundering is a series of steps involving sorting of articles to be laundered, their loading into and removal from the washer, drying, and then storage. Thus, best practices to prevent pathogen spread should involve both adequate processing of the clothes to remove pathogens, but also disinfecting any surfaces in contact with contaminated laundry or hands and good hand hygiene to prevent transmission to the person performing these tasks. Figure 1 shows the steps involved in processing home laundry.

### Removal of pathogens by laundering

Laundry detergents are primarily composed of builders (chelating or sequestering agents) to soften the hard water and surfactants (both ionic and anionic) that are responsible for the cleaning performance of the product. They may also include enzymes and other additives to improve the performance and appearance of fabrics after washing. For our purposes, we mark the distinction between:

- *Cleaning*: achieved via the combined action of laundry detergent ingredients, water and agitation to physically remove pathogens and other microbes, plus soils, stains and dirt from fabrics.
- *Sanitization and Disinfection*: achieved via antimicrobial chemicals proven to inactivate microorganisms and which are regulated by the US Environmental Protection Agency (EPA).

Removal of pathogens from the laundry is largely dependent on washing and drying practices. The reduction of pathogens is influenced by detergent selection, other additives (chlorine bleach), water temperature and drying. Relative to wash water temperature, the greatest risk may be in the US where 44.7% of households wash over 50% of their laundry loads on a cold‐water setting (Procter & Gamble unpublished internal data based on *n* = 54,136 loads of laundry done in 304 households in the US from 2018 to 2019). A cold‐water setting is defined as 16 ± 4.2℃ (AATCC Committee RA^88^, ^2017^). The median cold‐water wash in households in the United States is 14.4℃ (57.9°F), while hot water taps are recommended to be set at a maximum of 49℃ (120°F) to 52℃ (125°F) to avoid scalding (George, 2009). SARS‐CoV‐2 is reduced by greater than 4 logs after 5 min at 65℃ (149°F) and 20 min at 60℃ (140°F) (Abraham et al., 2020).

Although some enveloped viruses, such as SARS‐CoV‐2 and influenza, and Gram‐negative bacteria may survive high wash water temperatures, they are relatively sensitive to the action of detergents, which can eliminate such organisms even in median cold‐water wash conditions. Enteric viruses, and some bacteria and fungi, however, may require hot water washes with chlorine bleach, and high settings on dryers to achieve targeted reductions (Gerba & Kennedy, 2007; Heinzel et al., 2010). Heinzel et al. (2010) found that while enveloped viruses were inactivated by >99.99% by washing textiles at 20℃, temperatures of 30–40℃ along with a sanitizing detergent (activated oxygen) were necessary for the nonenveloped viruses (Heinzel et al., 2010). Both chlorine bleach and activated oxygen sanitizers result in a greater reduction of pathogens in textiles (Shin et al., 2020; Gerba, unpublished). However, caution should be used with chlorine bleach as it can damage many synthetic and synthetic‐natural blend textiles. Machine‐drying also provides an additional barrier, with both temperature and duration playing a role. Drying is recognized as an inextricably linked step in the washing process that significantly reduces germ load on fabrics (Brands et al., 2016). Brands et al. (2016) evaluated a variety of nonpathogenic and opportunistic bacteria and fungi and their log reduction factors (ranging from 1 to 4 log_10_) following different drying methods. Higher temperature settings and length of drying can significantly reduce microbial numbers (Munk et al., 2001; Gerba, unpublished).

Although many environmental and intrinsic pathogen factors impact microbial survival, few have been quantitatively evaluated relative to the risk of infections from domestic laundry. To our knowledge, this is the first study to utilize a QMRA approach to evaluate variable household wash conditions relative to log_10_ reductions of laundry pathogens and target infection risks over a wide range of situational scenarios (e.g. presence of ill or immunocompromised household members) and a wide range of pathogen types, including representative respiratory and enteric viruses and bacteria. We further applied these QMRA results to the development of situational guidelines to mitigating risks from pathogens in laundry under typical domestic practices.

## MATERIALS AND METHODS

### Risk assessment of the laundering process

We utilized a QMRA modelling framework for evaluating infection risks associated with handling contaminated clothing. The four‐step QMRA paradigm includes (1) hazard identification; (2) exposure assessment; (3) dose–response assessment and (4) risk characterization. Based on laundry processing steps, consideration of potential risks included both the person handling the laundry as well as the ability of the washing/drying process to reduce pathogen concentrations in the laundered material.

### Hazard identification

Little data are available reporting the concentration of pathogens in laundry. Die‐off, either naturally or with the application of targeted interventions and dilution factors, can result in reduced exposure and risk. On the other hand, some pathogens may increase in concentration via regrowth capabilities, particularly in moist or humid storage conditions and laundry heavily contaminated with bodily fluids. Here, we evaluate microbial hazards in laundry representative of a nonenveloped respiratory virus (rhinovirus), a nonenveloped enteric virus (rotavirus) and a Gram‐negative bacterium (nontyphoidal *Salmonella*) and estimate from the literature initial concentrations of 10^7^, 10^11^ and 10^10^ respectively (Table 3) (Gerba, 2000; L’Huillier et al., 2015). Die‐off during storage alone may be greater than 4 logs for unwashed laundry contaminated with enveloped or respiratory viruses (Gerhardts et al., 2016; Harbourt et al., 2020; Sakaguchi et al., 2010). Enteric pathogens, however, may survive well during room temperature storage, resulting in potentially high exposure levels from initial handling (Sattar et al., 1986).

**TABLE 3:** Parameters associated with laundry QMRA

Variable description	Units	Point estimate	Source/reference
Pathogen concentration			
Respiratory virus (rhinovirus)	log gc/ml secretions	7	L’Huillier et al. (2015)
Non‐enveloped virus	log CFU/g faeces	11	Gerba (2000)
Enteric bacteria	log CFU/g faeces	10	Gerba (2000)
Clothing stored at room temperature (24 h)			
Enveloped virus	Log reduction	4.5	Gerhardts et al. (2016)
Nonenveloped virus		0	Sattar et al. (1986)
Enteric bacteria		0	Sattar et al. (1986)
Contact with porous surface	Contacts/min	5.5	Beamer et al. (2015)
Transfer rate to hands:			
Virus/bacteria	Probability	0.003	Rusin et al. (2002); Lopez et al. (2013)
Face/orifice contact, adult	Contact	1	Assumed single event (Wilson et al., 2021)
Transfer rate to mouth	Rate/event	0.339	Rusin et al. (2002)
Cold water wash			
Enveloped virus (20℃)	Log reduction	>4	Heinzel et al. (2010)
Nonenveloped virus (20–23℃)		2.88	Gerba and Kennedy (2007)
Enteric bacteria (20–23℃)		2.1	Gerba et al. (2016)
Hot water wash:			
Enveloped virus (56℃)		>4	Abraham et al. (2020)
Non‐enveloped virus (54–60℃)		5.6	Sidwell et al. (1967)
Enteric bacteria (52℃)		>6.4	Honisch et al. (2014)
Regular detergent wash:			
Enveloped virus	Log reduction	>6	Gerhardts et al. (2016)
Nonenveloped virus		1.75	Kennedy and Gerba (1998)
Enteric bacteria		0.95	Gibson et al. (1999)
Advanced detergent wash			
Enveloped virus	Log reduction	>6.4	Honisch et al. (2014)
Nonenveloped virus		5.43	Kennedy and Gerba (1998)
Enteric bacteria		3	Gibson et al. (1999)
Chlorine bleach rinse			
Enveloped virus	Log reduction	>6	Assumed (Gerhardts et al., 2016)
Nonenveloped virus		4.52	Gibson et al. (1999)
Enteric bacteria		4–5	Bloomfield et al. (2013)
Machine drying			
Enveloped virus	Log reduction	1–2	Harbourt et al. (2020)
Nonenveloped virus		0.32	Kratzel et al. (2020)
Enteric bacteria		4.83	Gerba et al. (2016)
Alcohol‐based hand rub intervention			
Respiratory virus	Log reduction	6	Bloomfield et al. (2007)
Nonenveloped virus		4.6	Bloomfield et al. (2007)
Enteric bacteria		4.7	Bloomfield et al. (2013)
Pathogen dose–response parameters:			
Rhinovirus	Beta Poisson	α = 2.21E‐01	QMRAwiki.org
		β = 1.81E+00	
Rotavirus	Beta Poisson	α = 2.53E‐01	
		β = 6.17E+00	
*Salmonella*	Beta Poisson	α = 2.10E‐01	
		β = 4.98E+01	

### Exposure assessment

The risk assessment scenario involved a single user and a single exposure event at each step in the laundering process where the user was in direct contact with the laundry. Opportunities for hand transfer of microbes were considered during sorting of the laundry, transfer of washed laundry from the washing machine to a dryer or hang drying, and final storage or use (Sattar et al., 2001). Based on previous surface contact frequency data, we assumed 5.5 contacts/minute for a single laundry transfer event either to the washer or to the dryer (Beamer et al., 2015). Face contacts have been observed to occur at a rate of 0.33 per minute (Wilson et al., 2021) and transfer rates from hand‐to‐mouth at 0.339 per event. Here we assumed a single face‐touching event during the loading of laundry in the washer or during the transfer of wet laundry to the dryer. Additional parameters associated with reduced pathogen concentrations in laundry include rinse dilutions, hot vs. cold water washes, advanced and regular detergents, and the use of sanitizing products. Advanced detergents are defined as those that contain multiple surfactants and include enzymes. The efficacy of these interventions has been quantified in various studies and utilized as point estimates in our risk assessment calculations (Table 3).

Finally, we incorporated a hand hygiene intervention step at each discrete opportunity for a hand contamination event to occur after handling laundry. We included values for alcohol‐based hand rub efficacies for respiratory and enteric viruses, as well as enteric bacteria (Table 3).

### Dose–response assessment

Recommended dose–response parameters are compiled from the published literature and centrally posted on the QMRA wiki (QMRAwiki.org). The QMRA Wiki serves as a community resource for dose–response parameters from peer‐reviewed publications to be used in quantitatively linking exposures to a known dose of a specific pathogen. Using this information, we calculated the probability of adverse response, such as infection. Table 3 summarizes variables and parameter values associated with initial pathogen concentrations in laundry, human exposure potentials and pathogen‐specific dose–response information that contributes to the laundry risk characterization. Currently, there are limited data and primarily only point estimates available in the literature on QMRA parameters for quantifying domestic laundry‐transmitted infection risks. Wide variability in log_10_ reductions for different parameters is also evident due to a lack of standardization of experimental test conditions.

### Risk characterization

We evaluated exposure scenarios and associated dose concentrations using single‐point estimates of most likely values to get a sense of the sensitivity of different input variables and assumptions. Given that published empirical data specific to laundry practices are limited and sometimes conflicting or overly simplified, simple point value inputs are often the only data available but are still useful to explore changing scenarios or intervention impacts. Event tree scenarios are presented for select representative respiratory and enteric viruses and bacteria. We compared estimated infection risks to an acceptable risk threshold of 1.0 × 10^−6^ infections per person per event. While no standard regulations or guidelines are available defining acceptable risks in household laundry applications, we based our target on drinking water regulatory standards of 1.0 × 10^−4^ and added a 100‐fold safety factor to be more protective of immunocompromised populations. This approach has been used in previous QMRA studies for evaluating surface disinfection efficacies against microbial pathogens to estimated health outcomes (Wilson et al., 2020).

## RESULTS

Table 4 details the QMRA results for rhinovirus, rotavirus and *Salmonella* pathogens. For rhinovirus, all scenarios of washing, when combined with detergent use, achieved a risk approaching or exceeding the acceptable infection risk target of 1 in a million (1.0 × 10^−6^) per event. The addition of a hand hygiene intervention following contamination of the hands and before a face/orifice contact reduced risks to very low levels (e.g. >2.16 × 10^−11^) for all wash scenarios. In our calculations, we evaluated hand hygiene efficacies of 6, 4.6 and 4.7 log_10_ reductions from alcohol‐based hand sanitizers as reported in the literature. These efficacy values, however, are subject to uncertainty and should be experimentally measured in laundry practice scenarios.

**TABLE 4:** QMRA laundry single user event scenarios

Event tree—nonenveloped, respiratory virus, rhinovirus		Wash		Detergent		Other			
Original concentration	Die‐off during hamper storage	Cold	Hot	Regular	Advanced	Machine dry/cold wash	Chlorine bleach rinse/cold wash	Machine dry/hot wash	Chlorine bleach rinse/hot wash
1.00E+07	3.16E+02	3.16E‐02	3.16E‐02	3.16E‐04	1.26E‐04	3.16E‐03	3.16E‐08	3.16E‐03	3.16E‐08
Transfer to hands	5.22E+00	5.22E‐04	5.22E‐04	5.22E‐06	2.08E‐06	5.22E‐05	5.22E‐10	5.22E‐05	5.22E‐10
Transfer to face/orifice	1.77E+00	1.77E‐04	1.77E‐04	1.77E‐06	7.04E‐07	1.77E‐05	1.77E‐10	1.77E‐05	1.77E‐10
Dose concentration	1.77E+00	1.77E‐04	1.77E‐04	1.77E‐06	7.04E‐07	1.77E‐05	1.77E‐10	1.77E‐05	1.77E‐10
*Infection risk (single event)*	1.40E‐01	2.16E‐05	2.16E‐05	2.16E‐07	8.60E‐08	2.16E‐06	2.16E‐11	2.16E‐06	2.16E‐11
Transfer to face/orifice w/hand hygiene intervention	1.77E‐06	1.77E‐10	1.77E‐10	1.77E‐12	7.04E‐13	1.77E‐11	1.77E‐16	1.77E‐11	1.77E‐16
Dose concentration w/hand hygiene intervention	1.77E‐06	1.77E‐10	1.77E‐10	1.77E‐12	7.04E‐13	1.77E‐11	1.77E‐16	1.77E‐11	1.77E‐16
*Infection risk w/hand hygiene intervention*	2.16E‐07	2.16E‐11	2.16E‐11	2.16E‐13	8.59E‐14	2.16E‐12	0.00E+00	2.16E‐12	0.00E+00

Event tree—nonenveloped enteric virus, rotavirus		Wash		Detergent		Other			
Original concentration	Die‐off during hamper storage	Cold	Hot	Regular	Advanced	Machine dry/cold wash	Chlorine bleach rinse/cold wash	Machine dry/hot wash	Chlorine bleach rinse/hot wash
1.00E+11	1.00E+11	1.32E+08	2.51E+05	1.78E+09	3.72E+05	6.31E+07	3.98E+03	1.20E+05	7.59E+00
Transfer to hands	1.65E+09	2.18E+06	4.14E+03	2.93E+07	6.13E+03	1.04E+06	6.57E+01	1.98E+03	1.25E‐01
Transfer to face/orifice	5.59E+08	7.37E+05	1.41E+03	9.95E+06	2.08E+03	3.53E+05	2.23E+01	6.72E+02	4.24E‐02
Dose concentration	5.59E+08	7.37E+05	1.41E+03	9.95E+06	2.08E+03	3.53E+05	2.23E+01	6.72E+02	4.24E‐02
*Infection risk (single event)*	9.90E‐01	9.48E‐01	7.47E‐01	9.73E‐01	7.71E‐01	9.37E‐01	3.21E‐01	6.96E‐01	1.73E‐03
Transfer to face/orifice w/hand hygiene intervention	1.41E+04	1.85E+01	3.53E‐02	2.50E+02	5.22E‐02	8.87E+00	5.59E‐04	1.69E‐02	1.07E‐06
Dose concentration w/hand hygiene intervention	1.41E+04	1.85E+01	3.53E‐02	2.50E+02	5.22E‐02	8.87E+00	5.59E‐04	1.69E‐02	1.07E‐06
*Infection risk w/hand hygiene intervention*	8.59E‐01	2.96E‐01	1.44E‐03	6.10E‐01	2.13E‐03	2.02E‐01	2.29E‐05	6.91E‐04	4.37E‐08

Event tree—enteric bacteria, *Salmonella* spp.		Wash		Detergent		Other			
Original concentration	Die‐off during hamper storage	Cold	Hot	Regular	Advanced	Machine dry/cold wash	Chlorine bleach rinse/cold wash	Machine dry/hot wash	Chlorine bleach rinse/hot wash
1.00E+10	1.00E+10	7.94E+07	3.98E+03	1.12E+09	1.00E+07	1.17E+03	7.94E+03	5.89E‐02	3.98E‐01
Transfer to hands	1.65E+08	1.31E+06	6.57E+01	1.85E+07	1.65E+05	1.94E+01	1.31E+02	9.72E‐04	6.57E‐03
Transfer to face/orifice	5.59E+07	4.44E+05	2.23E+01	6.28E+06	5.59E+04	6.57E+00	4.44E+01	3.29E‐04	2.23E‐03
Dose concentration	5.59E+07	4.44E+05	2.23E+01	6.28E+06	5.59E+04	6.57E+00	4.44E+01	3.29E‐04	2.23E‐03
*Infection risk (single event)*	9.46E‐01	8.52E‐01	7.47E‐02	9.15E‐01	7.71E‐01	2.57E‐02	1.25E‐01	1.39E‐06	9.39E‐06
Transfer to face/orifice w/hand hygiene intervention	1.12E+03	8.87E+00	4.44E‐04	1.25E+02	1.12E+00	1.31E‐04	8.87E‐04	6.57E‐09	4.44E‐08
Dose concentration w/hand hygiene intervention	1.12E+03	8.87E+00	4.44E‐04	1.25E+02	1.12E+00	1.31E‐04	8.87E‐04	6.57E‐09	4.44E‐08
*Infection risk w/hand hygiene intervention*	4.84E‐01	3.38E‐02	1.87E‐06	2.32E‐01	4.64E‐03	5.53E‐07	3.74E‐06	2.77E‐11	1.87E‐10

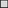
 meets acceptable risk threshold of ≥1.0 × 10^−4^; 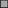 meets acceptable risk threshold of ≥1.0 × 10^−6^.

Previous studies, and our QMRA, confirm that enteric viruses, such as rotavirus, present worst‐case scenarios and very high‐risk estimates, ranging from 9.48 × 10^−1^ to 1.73 × 10^−3^ for all wash parameters, from exposure to contaminated laundry. This is due to their low infectious dose and ability to survive following laundry washing and drying interventions (Gerba & Kennedy, 2007; Lemm et al., 2014). For rotavirus, acceptable risk limits were only achieved following the use of hot water, advanced detergents, chlorine bleach sanitizers and implementation of a hand hygiene intervention before face/orifice contact (e.g. 4.37 × 10^−8^).

Laundry contaminated with *Salmonella* also presents unacceptable risks over most scenarios except for hot water washes combined with drying or chlorine bleach use where the 1.0 × 10^−6^ threshold was nearly achieved (1.39 × 10^−6^ and 9.93 × 10^−6^ respectively). Adding a hand hygiene intervention achieved or approached acceptable risk levels when laundry was washed in hot water (1.87 × 10^−6^) or when cold or hot wash water interventions were combined with machine drying (5.53 × 10^−7^ and 2.77 × 10^−11^ respectively) or with the use of a chlorine bleach sanitizer (1.87 × 10^−10^).

Our single user scenario shows that acceptable risk targets are easily achieved relative to respiratory pathogens under North American wash conditions using cold water washes (e.g. median temperature 14.4℃) with regular detergents and drying. Representative enteric virus and bacterial pathogens, however, require a more aggressive intervention to reduce risks, including the use of hand hygiene at critical control points (after transfer of laundry to washer or dryer and before touching the face) to achieve acceptable risk limits.

## DISCUSSION

A variety of host, environmental and pathogen‐specific factors play a role in estimating the probability of being exposed to an environmental pathogen and subsequent infection and illness. Exposure assessment requires consideration of initial concentrations of pathogens in the textiles, as well as specific scenarios that impact contact frequencies and dose concentrations. For example, in the case of laundry, pathogen or environment‐specific persistence factors and human behaviours that drive exposure and dose concentrations may include how soiled laundry is handled and stored, textile composition, type of laundry detergents or sanitizers used, wash water temperature and drying processes. Other household members in contact with surfaces contaminated by soiled laundry are another uncontrolled source of pathogen transmission where the targeted use of surface disinfectants may help to reduce environmental exposure risks. In addition, recommendations for shared domestic laundry facilities, such as within apartment buildings, where laundry contamination and cross‐contamination potentials from prior users are unknown, include a higher level of infection control strategies.

Transfer of the microorganism from the textiles to the hands may result in cross‐contamination and transfer to the mouth, eyes, or nose resulting in infection. Here we utilize previously published observational data to estimate infection risks for a single contact with the face and susceptible orifices and to calculate the concentration of pathogen transferred to the hands (Wilson et al., 2021). Textile material types may also play a role in the transfer of pathogens. For example, 100% cotton fabrics compared to synthetic mixtures (50:50% cotton/polyester) may have a 10‐fold or higher rate of transfer to the hands (Rusin et al., 2002). Microbial transfer from textiles is much less (<1%) than from hard surfaces such as plastics and steel surfaces (30%–70%) (Lopez et al., 2013; Rusin et al., 2002) however, the type of material and degree of soiling and moisture could influence the degree of transfer.

Limitations of our QMRA study include uncertainty in the input variables, such as the initial concentrations of pathogens in the laundry and washing machine, and detergent or sanitizer efficacy, duration of wash and dry cycles, ranges in pathogen survival and transfer, and human behaviours in terms of contact frequency specifically related to handling contaminated laundry and face‐touching opportunities. Inconsistency across studies revealed gaps in intervention efficacy studies.

In addition, real‐world exposure scenarios may involve multiple users and an increased risk of infection among multiple household members through both direct contact with contaminated laundry and indirect contact through household surfaces that are also directly or indirectly contaminated from laundry sources. Such a risk characterization would need input data representative of the complex, interactive behaviours and contact sequences for each family member along with an understanding of the related exposure assessment variables associated with those contacts. To date, such quantitative exposure information is not available in the literature. Although our models and guidelines do not address multiple user scenarios, they remain useful to explore changing laundry conditions and intervention impacts.

Future studies could analyse a broader set of data using stochastic variable inputs instead of point estimates, however for most variables listed, stochastic data are not available. There is a need for the collection of empirical data on the specific input variables of our QMRA model and to apply more sophisticated efforts in probabilistic risk assessment where uncertainty in these values can be evaluated over a range of more complex distributions. In addition, more real‐world observational data are needed to advance the risk model beyond the single user context of the current paper toward multiple user scenarios.

### Strategy for home laundering

Based on our QMRA and current CDC guidelines for handling laundry potentially contaminated with pathogens, we have compiled recommended strategies for home laundering (Table 5) (CDC, 2020). In the absence of illness in household members, a scenario of using regular detergent and/or cold‐water washes is expected to be sufficient for the removal of nonpathogenic, indigenous microbes that are known to pose minimal health risks in immunocompetent persons. Households with healthy, active persons associated with heavier staining and body soiling can benefit from the use of higher‐quality detergents containing multiple types of surfactants and enzymes to remove soils deeply embedded within textiles.

**TABLE 5:** Recommended strategies for home laundering

	Level of laundry hygiene and/or sanitization			
Situation	Healthy households with light staining and bodily soiling	Healthy households with heavy staining and bodily soiling	Households with suspected or confirmed respiratory infections including COVID‐19, influenza or the common cold	Households with confirmed or suspected enteric infections (‘stomach bugs’) Households with persons who have a weakened immune system Households with healthcare workers and first responders; laundering work clothes at home
General guidance	Standard laundry process using quality detergents provides adequate hygiene for every‐day laundering	Active households with heavier soiling can benefit from higher quality detergents (characterized by multiple types of surfactants and enzymes) to deeply clean stains and body soil residues from textiles	Special precautions should be taken when handling contaminated clothes and bedding as per the CDC guidelines, but sanitizers are not needed to remove respiratory viruses	Sanitizers and/or the sanitizing cycle on the washing machine should be used during certain illness or special situations, but they should be used in combination with higher quality detergents to first remove deeply embedded soils, and should not be used for everyday cleaning
Special handling precautions	Wash hands after handling soiled laundry and transferring wet laundry from washer to dryer; avoid contact between contaminated surfaces and soiled laundry	Wash hands after handling soiled laundry and transferring wet laundry from washer to dryer; avoid contact between contaminated surfaces and soiled laundry	*Wear disposable gloves* when handling dirty laundry (clothes and bed linens) from a person who is sick; Dirty laundry from a person who is sick *can be washed with other people's items. Do not shake dirty laundry; Clean and disinfect clothes hampers and contact surfaces*; Remove gloves, and *wash hands immediately*; Wash hands again after transferring wet laundry from washer to dryer	Work clothes should be removed before entering the domestic environment; *Wear disposable gloves* when handling dirty laundry (clothes and bed linens) from a person who is sick**;** Dirty laundry from a person who is sick *should not be washed with other people's items; Do not shake* dirty laundry; clean and *disinfect clothes hampers and contact surfaces*; Remove gloves, and *wash hands right away*; wash hands again after transferring wet laundry from washer to dryer
Recommended products	Regular (low surfactant; no enzymes) or high quality (high surfactant; enzymes) laundry detergent	Higher quality detergents (multiple types of surfactants and enzymes)	High or higher quality detergents	*High‐quality laundry detergent PLUS Registered Sanitizer or Sanitizing Cycle* on the washing machine
Washing frequency	Wash as needed	Wash frequently	Wash as soon as possible	Wash as soon as possible
Washer settings	Regular detergent: Cold water for most clothes; use warm/hot water for socks, underwear, sheets, and towels, and cleaning cloths; high‐quality detergent: wash at any temperature, including cold water	Wash at any temperature, including cold water	Use the warmest water setting allowed by the care label	*Hottest water allowed* by the care label; follow *registered sanitizer* product instructions or washing machine instructions for the sanitizing cycle
Dryer setting	Medium heat; allow clothes to dry completely before storing	Low to medium heat; allow clothes to dry completely before storing	Medium to high heat; dry completely before storing	*Highest heat setting* allowed by the care label; dry completely before storing

Increased shading is used to differentiate levels of response with darker shading indicating a higher level of hygiene controls needed.

Households with suspected or confirmed respiratory infections, including COVID‐19, influenza or colds should use special precautions when handling contaminated laundry items but can achieve acceptable risk targets using high‐quality detergents without special additives or separating laundry items from ill family members (Table 5). That is because all of these respiratory infections are caused by enveloped viruses, like SARS‐CoV‐2, which are very sensitive to the types of surfactants found in high‐quality laundry detergents (Jahromi et al., 2020).

Households with clothing from individuals with enteric illnesses caused by non‐enveloped enteric viruses, such as rotavirus, should consider more effective interventions, such as elevated temperature for washing and drying and the use of chlorine bleach or registered sanitizing additives (Table 5). That is because they are more resistant to the action of detergents and drying (Boone & Gerba, 2007; Gerba & Kennedy, 2007; Lemm et al., 2014). Children may experience three diarrhoeal and six to 12 respiratory infections per year, however, this number can be highly variable. These precautions should also be considered when laundering certain professional clothing where broad pathogen contamination potentials exist, including the highly resistant spore‐forming bacteria such as *Clostridiodes difficile* (Tarrant et al., 2018). Examples include work clothes from individuals employed in healthcare, wastewater, agriculture and food processing industries. In the case of enteric illnesses, consideration should also be given to processing clothing separately from ill individuals and professional clothing from other household clothing to reduce the possibility of cross‐contamination of other washed laundry (Callewaert et al., 2015; Nordstrom et al., 2012). Contaminated clothing from these households should also be washed and dried at the hottest temperatures allowed without damaging the items in question as heat can play a role in inactivating pathogenic microorganisms (Bockmühl et al., 2019; Riley et al., 2017). One study found that the greatest drying log reduction for a range of bacteria and fungi was with clothesline outside drying methods, where natural UV light exposure from the sun aided in microbial reductions. Reductions for *Aspergillus niger* spores, however, were approximately 1 log_10_ regardless of drying method (Brands et al., 2016).

It is also important to emphasize good hand hygiene practices (handwashing or use of an alcohol‐based hand sanitizer) during each step in the laundry process as hands may become contaminated by the clothing, especially during transfer from soiled hampers and between the washer and dryer, resulting in unacceptable risk levels. Our study showed that proper hand hygiene practices implemented at each step of the laundry handling process were the most important and effective driver of risk reductions, however, hand hygiene alone does not meet acceptable risk targets for all scenarios. Therefore, when handling clothing from individuals with respiratory or enteric infections, proper use of disposable gloves is advised to reduce the risk of hand contamination in addition to hand hygiene interventions (Table 5).

Based on the results of this single user scenario study, we conclude that domestic laundering practices are important in reducing the risk of infection in households. In ‘healthy’ households, the risk of infection from washed clothes is estimated to be extremely low. In such households, the standard laundry process combined with a quality laundry detergent is expected to meet target risk reductions, even in median cold‐water wash temperature conditions (e.g. 14.4℃), and especially when combined with washing or sanitizing hands after handling or transferring soiled or wet laundry. The use of higher quality detergents, characterized by multiple types of surfactants and enzymes are recommended to deeply clean stains and bodily soil residues or with specific types of laundry subject to heavy microbial contamination (e.g. diapers, undergarments and towels).

In households with ill or more susceptible individuals (e.g. infants, elderly, weakened immune systems), a strategy must be developed for laundry handling. Laundering strategies in the case of illness or special situations are further dependent on the type of pathogen associated with the illness. For example, respiratory infections caused by enveloped viruses, like SARS‐CoV‐2, elicit special laundry handling precautions but do not require special washing conditions, such as the use of registered sanitizers. Illnesses caused by enteric viruses and households with at‐risk individuals or high occupational exposures to pathogens, however, require additional options for achieving acceptable risk limits, such as higher wash and drying temperatures, and the use of chlorine bleach or other registered sanitizers. Sanitizers should be used after washing with a higher quality detergent to remove soils that can interfere with sanitizer efficacy. Finally, to meet acceptable risk targets, good hand hygiene should always be practiced when handling household laundry, and the use of gloves is advised when there are ill individuals in the home.

## CONFLICT OF INTEREST

Funding was provided, in part, via an unrestricted gift from The Procter & Gamble Corporation.
